# Single-photon transistor based on cavity electromagnetically induced transparency with Rydberg atomic ensemble

**DOI:** 10.1038/s41598-019-41185-2

**Published:** 2019-03-18

**Authors:** Y. M. Hao, G. W. Lin, X. M. Lin, Y. P. Niu, S. Q. Gong

**Affiliations:** 1 Department of Physics, East China University of Science and Technology, Shanghai, 200237 China; 2 College of Physics and Energy, Fujian Normal University, Fuzhou, 350108 China

## Abstract

A scheme is presented to realize a single-photon transistor based on cavity quantum electrodynamics (QED) with Rydberg atomic ensemble. By combining the advantages of the cavity-enhanced interaction and Rydberg blockade, we achieve a high gain single-photon transistor. The numerical calculation shows that by using one single gate photon more than one thousand source photons can be switched.

## Introduction

Electromagnetically induced transparency (EIT) induced by the coherent interference effect has many important applications, including optical nonlinearities^1,2^, quantum storage^3,4^, observation of parity-time symmetry^5–7^, and so on^8,9^. By combining the advantages of the cavity-enhanced interaction and Rydberg blockade, Cavity EIT with Rydberg atomic ensemble becomes a promising platform for the realization of optical nonlinearities. Both theories and experiments^10–13^ have verified strong optical nonlinearities can be realized in this system. In particular, Lin *et al*.^14^ presented a theoretical scheme for strong single-photon nonlinearity with intracavity EIT in blockaded Rydberg ensemble. In this scheme, they showed that the photons in the cavity are in the form of cavity dark-state polaritons, and strong interaction of the polaritons leads to strong blockade effect. In a recent experiment^15^, Jia *et al*. have observed this strong interaction of the cavity dark-state polaritons, and demonstrated the strong single-photon nonlinearities by measuring the transmission spectrum. By exploiting this strong nonlinearities, the quantum phase gate between a photon and an atomic ensemble^16^ or two photons^17^ can be realized.

Single-photon transistor is the cornerstone device for quantum information processing^18–22^. It opens up new perspectives for all-optical information processing and has many potential applications, such as realization of quantum repeaters^23^, nondestructive detection of optical photons^24^, generation of Schrödinger-cat states^25^. Strong single-photon nonlinearities, by which a gate light pulse changes the transmission of a source light pulse with a gain above unity, are the fundamental limit of such devices. Much efforts towards obtaining such strong single-photon nonlinearities have been made in various systems^26–32^. Among these systems, cavity quantum electrodynamics (QED)^33^ and Rydberg EIT^34–39^ are two significant promising candidates. To obtain a high gain single-photon transistor, the cavity QED scheme^33^ and the Rydberg-EIT scheme^38^ respectively use the cavity-enhanced interaction and Rydberg blockade^40,41^ to achieve the strong single-photon nonlinearities. Both optical gain of these two systems have been up to several hundreds^33,38^.

In this paper, we theoretically present a single-photon transistor based on cavity EIT with Rydberg atomic ensemble^42,43^. In our scheme, a Rydberg atomic ensemble is trapped in an optical cavity. The extremely strong single-photon nonlinearities can be created by combining the advantages of the cavity-enhanced interaction and Rydberg blockade. By means of the strong single-photon nonlinearities, we can implement a single-photon transistor with high gain. We show that the optical gain in our scheme could be boosted above one thousand, which is higher than that in both cavity QED scheme^33^ and Rydberg-EIT scheme^34–38^. Furthermore, even when the scale of atomic ensemble is much larger than the blockade radius^44^, our scheme could still work well.

## Results

**Figure 1:**
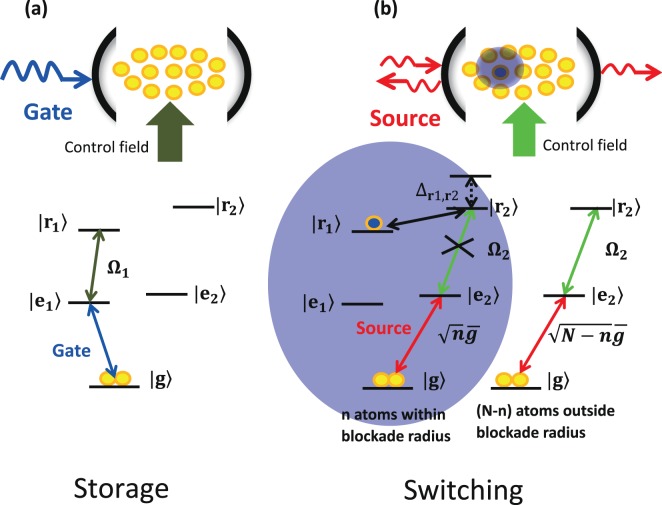
Single-photon transistor with an ensemble of N Rydberg atoms trapped inside an optical cavity. (**a**)We first stored a gate photon in the medium, which corresponds to a Rydberg excitation to state |*r*_1_〉. (**b**) This Rydberg excitation blocks the transmission of source photons through the cavity.

As illustrated in Fig. 1, our model consists of a cold ensemble of *N* Rydberg atoms trapped inside an optical cavity. Each atom has a stable ground state |*g*〉, two excited states |*e*_1_〉 and |*e*_2_〉, and two Rydberg states |*r*_1_〉 and |*r*_2_〉. The first step for realization of the single-photon transistor is the process of the gate photon. Initially, all atoms are in ground state |*g*〉, i.e., $|G\rangle =|{g}_{1},{g}_{2},\ldots ,{g}_{N}\rangle$. A free-space gate photon is resonant to the transition |*g*〉 ↔ |*e*_1_〉, while a classical control field with Rabi frequency Ω_1_ drives the transition |*e*_1_〉 ↔ |*r*_1_〉, as shown in Fig. 1(a). These two transitions form EIT configuration. By adiabatically changing the control laser power down, the gate photon can be stored in the Rydberg atomic ensemble^3^. As shown in ref. ^4^, the maximum storage efficiency with cavity EIT could reach *P* ≈ *Nη*/(1 + *Nη*) after optimized control, here *η* is the single-atom cooperativity. For *Nη* ≫ 1, *P* → 1.

After this storage process of the gate photon, we apply a weak coherent source beam *ε*_*s*_ with frequency *ω*_*s*_ to drive the cavity mode *a*, which is resonant with the transition |*g*〉 ↔ |*e*_2_〉, as seen in Fig. 1(b). Meanwhile another control field with Rabi frequency Ω_2_ drives the transition |*e*_2_〉 ↔ |*r*_2_〉. The total Hamiltonian of the system can be given by

1 $${H}_{total}={\varepsilon }_{s}({a}^{\dagger }{e}^{-i{{\rm{\Delta }}}_{s}t}+a{e}^{i{{\rm{\Delta }}}_{s}t})+{H}_{I},$$

with Δ_*s*_ = *ω*_*s*_ − *ω*_*a*_ being detuning of source photon from the cavity mode, *a* (*a*^†^) being the annihilation (creation) operator of the cavity mode, and

2 $$\begin{array}{rcl}{H}_{I} & = & \mathop{\sum _{j=1}}\limits^{N}({g}_{j}|{e}_{\mathrm{2,}j}\rangle \langle {g}_{j}|a+{{\rm{\Omega }}}_{2}(|{r}_{2,j}\rangle \langle {e}_{\mathrm{2,}j}|+H\mathrm{.}c\mathrm{.}\\  &  & +\sum _{i < j}{{\rm{\Delta }}}_{{r}_{1},{r}_{2}}(|{r}_{\mathrm{2,}j}\rangle \langle {r}_{\mathrm{2,}j}|\otimes |{r}_{\mathrm{1,}i}\rangle \langle {r}_{\mathrm{1,}i}|)\end{array}$$

being the interaction Hamiltonian between atoms and cavity mode. Here ${{\rm{\Delta }}}_{{r}_{1},{r}_{2}}$ is the additional energy shift when two atoms are respectively excited to Rydberg states |*r*_1_〉 and |*r*_2_〉, *g*_*j*_ is the single-atom cavity coupling coefficient. In Hamiltonian *H*_*I*_, we have ignored the self-blockade interaction ${\sum }_{i < j}{{\rm{\Delta }}}_{{r}_{2},{r}_{2}}(|{r}_{\mathrm{2,}j}\rangle \langle {r}_{\mathrm{2,}j}|\otimes |{r}_{\mathrm{2,}i}\rangle \langle {r}_{\mathrm{2,}i}|)$ by choosing suitable principal quantum number to satisfy ${{\rm{\Delta }}}_{{r}_{1},{r}_{2}}\gg {{\rm{\Delta }}}_{{r}_{2},{r}_{2}}$^45^.

In the ideal case, each atom has an equal probability-amplitude $\mathrm{1/}\sqrt{N}$ to be the Rydberg excitation |*r*_1_〉 after the gate photon is stored in the atomic ensemble. Without loss of generality, we assume the *i*-*th* atom to be the Rydberg excitation |*r*_1_〉 and other atoms remain in their initial ground state |*g*〉. Due to the Rydberg blockade, *n* atoms within the blockade radius around the *i*-*th* atom have an additional energy shift ${{\rm{\Delta }}}_{{r}_{1},{r}_{2}}$. The other (*N* − *n*) atoms outside the blockade radius will not be affected by the Rydberg blockade (see Fig. 1(b)). For simplicity, we divide the Hamilton *H*_*I*_ into two parts: one part is Hamilton *H*_*n*_ for *n* atoms within the Rydberg blockade radius, the other is Hamilton *H*_*N*−*n*_ for (*N* − *n*) atoms outside the Rydberg blockade radius, then

3 $${H}_{I}={H}_{n}+{H}_{N-n},$$

with

4 $${H}_{n}=\mathop{\sum _{t=1}}\limits^{n}[({g}_{t}|{e}_{\mathrm{2,}t}\rangle \langle {g}_{t}|a+{{\rm{\Omega }}}_{2}|{r}_{\mathrm{2,}t}\rangle \langle {e}_{\mathrm{2,}t}|+H\mathrm{.}c.\,)+{{\rm{\Delta }}}_{{r}_{1},{r}_{2}}|{r}_{\mathrm{2,}t}\rangle \langle {r}_{\mathrm{2,}t}|],$$

5 $${H}_{N-n}=\mathop{\sum _{k=1}}\limits^{N-n}({g}_{k}|{e}_{\mathrm{2,}k}\rangle \langle {g}_{k}|a+{{\rm{\Omega }}}_{2}(|{r}_{\mathrm{2,}k}\rangle \langle {e}_{\mathrm{2,}k}|+H.\,c.\,).$$

Considering photon losses from the cavity as well as the decays associated with the atom, the dynamics of the system governed by the Hamiltonian *H*_*total*_ can be described by quantum Langevin equations^46^ and the steady-state solution of the cavity mode *a* under the mean-field approximation^47^ is given by (see Methods)

6 $$\langle a\rangle =\frac{-i{\varepsilon }_{s}}{i{{\rm{\Delta }}}_{s}+\kappa +\frac{{\bar{g}}^{2}n}{i{{\rm{\Delta }}}_{s}+\frac{{\gamma }_{e}}{2}+\frac{{{\rm{\Omega }}}_{2}^{2}}{i{{\rm{\Delta }}}_{s}+i{{\rm{\Delta }}}_{{r}_{1},{r}_{2}}+\frac{{\gamma }_{r}}{2}}}+\frac{{\bar{g}}^{2}(N-n)}{i{{\rm{\Delta }}}_{s}+\frac{{\gamma }_{e}}{2}+\frac{{{\rm{\Omega }}}_{2}^{2}}{i{{\rm{\Delta }}}_{s}+\frac{{\gamma }_{r}}{2}}}},$$

where *κ* is the decay rate of the cavity, *γ*_*e*_ (*γ*_*r*_) is the dephasing rate associated with the low excited state (Rydberg state), $\bar{g}=\sqrt{{\sum }_{j=1}^{N}{g}_{j}^{2}/N}$ is the effect atom-cavity coupling strength. And the transmitted source field through the cavity is

7 $${a}_{T}=\sqrt{\kappa }\langle a\rangle .$$

**Figure 2:**
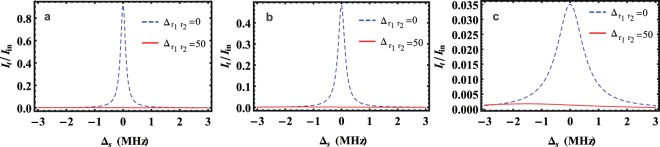
The transmitted intensity ${I}_{t}={a}_{T}^{\ast }{a}_{T}$ of the source light (normalized by the input source intensity ${I}_{in}={|{\varepsilon }_{s}|}^{2}$) versus the source-cavity detuning Δ_*s*_ with ${{\rm{\Delta }}}_{{r}_{1},{r}_{2}}=0$ MHz (blue dashed) and ${{\rm{\Delta }}}_{{r}_{1},{r}_{2}}=50$ MHZ (red solid) for (**a**) *γ*_*r*_ = 0.01*κ*, (**b**) *γ*_*r*_ = 0.1*κ* and (**c**) *γ*_*r*_ = 1*κ*. Other parameters are Ω = 20 MHz, *κ* = 1 MHz, $\bar{g}=1$ MHz, *N* = 3600, *n* = 400, *γ*_*e*_ = 30 MHz and *ε*_*s*_ = 5 MHz.

**Figure 3:**
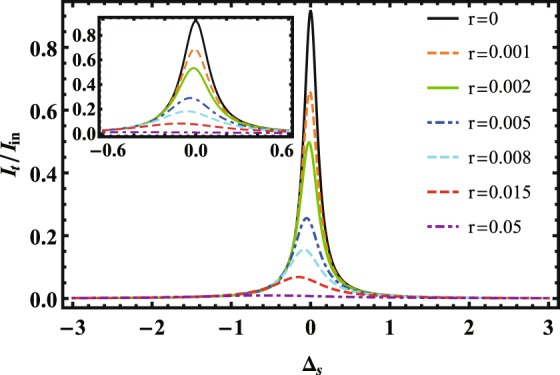
The normalized transmitted intensity of source-light vs source-cavity detuning with different values of r(n/N). Other parameters are Ω = 20 MHz, *κ* = 1 MHz, $\bar{g}=1$ MHz, *N* = 3600, *γ*_*e*_ = 30 MHz, *γ*_*r*_ = 10^−2^*κ*, ${{\rm{\Delta }}}_{{r}_{1},{r}_{2}}=50$ MHz and *ε*_*s*_ = 5 MHz.

Figure 2 plots the transmitted intensity ${I}_{t}={a}_{T}^{\ast }{a}_{T}$ of the source light (normalized by the input source intensity ${I}_{in}={|{\varepsilon }_{s}|}^{2}$) versus the source-cavity detuning Δ_*s*_ for (a) *γ*_*r*_ = 0.01*κ*, (b) *γ*_*r*_ = 0.1*κ*, (c) *γ*_*r*_ = 1*κ*. When ${\gamma }_{r}\ll \kappa$ and ${{\rm{\Delta }}}_{{r}_{1},{r}_{2}}=0$ MHz (i.e., no gate photon is stored into the Rydberg ensemble), we can observe a high transmission as all atoms form the EIT. When ${{\rm{\Delta }}}_{{r}_{1},{r}_{2}}\ne 0$ MHz (i.e., the gate photon has been stored as the Rydberg excitation), the transmission of the source beam will be suppressed greatly. Figure 3 depicts the normalized transmitted intensity of the source light as the function of the source-cavity detuning Δ_*s*_ with different values of ratio *r* = *n*/*N*. Quite unexpectedly, even though *r* ≪ 1, i.e., many atoms outside the blockade radius, the single Rydberg excitation can also block the transmission of source-light.

The physical reasons for the results above are as follows: when there are no stored gate photon, almost all source photons satisfy the EIT condition and are in dark states (energy eigenvalues *E*_0_ = 0). Then the energy of the cavity and atoms are $E={\omega }_{c}{a}^{\dagger }a+{E}_{0}={\omega }_{c}{a}^{\dagger }a$. In this case, the source photons will pass through the cavity when they resonate with the cavity mode. When the gate photon is stored in the ensemble, the coupling between the atoms and the cavity mode can be divided into two parts (within the blockade radius and outside the blockade radius). Atoms outside the blockade radius still satisfy the EIT and all are in dark states. The energy of this part is *E*_*N*−*n*_ = 0. But atoms within the blockade no longer satisfy the EIT condition due to the shift of the Rydberg energy level. The energy of this part is ${E}_{n}\ne 0$ since they have deviated from the dark state. Thus the total energy of the system $E={\omega }_{c}{a}^{\dagger }a+{E}_{N-n}+{E}_{n}\ne {\omega }_{c}{a}^{\dagger }a$. In this case, the source photons will be reflected by the cavity since they do not match the energy of the cavity-atom system.

Through the analysis above, a Rydberg excitation associated with the storage of one gate photon can suppress the transmission of the source photon, hence our model can be used to implement a single-photon transistor.

## Discussion and Conclusions

**Figure 4:**
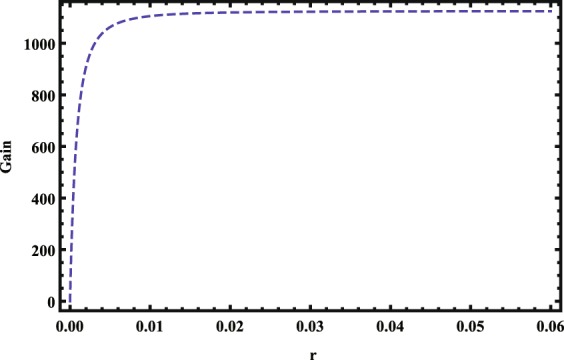
The transistor gain G versus r. Other parameters are Ω = 20 MHz, *κ* = 1 MHz, *N* = 3600, *γ*_*e*_ = 30 MHz, *γ*_*r*_ = 10^−2^*κ*, $\eta ={\mathop{g}\limits^{-}}^{2}/\kappa {\gamma }_{e}=0.1$, ${{\rm{\Delta }}}_{{r}_{1},{r}_{2}}=50$ MHz, *ε*_*s*_ = 5 MHz and *τ* = 50 *μ*s.

Next we quantify the single-photon transistor with optical gain. The transmitted source photon number is ${\bar{M}}_{s,\mathrm{out}}={\int }_{0}^{\tau }{a}_{T}^{\ast }{a}_{T}dt$ with *τ* being the source integration time. Then the optical gain *G* per stored gate photon can be defined as the gate-photon-induced change in source-light transmission^33^, as

8 $$G={\bar{M}}_{s,\mathrm{out}}^{{\rm{no}}\,{\rm{gate}}}-{\bar{M}}_{s,\mathrm{out}}^{{\rm{with}}\,{\rm{gate}}},$$

where ${\bar{M}}_{s,\mathrm{out}}^{{\rm{no}}\,{\rm{gate}}}$ and ${\bar{M}}_{s,\mathrm{out}}^{{\rm{with}}\,{\rm{gate}}}$ denote the mean numbers of transmitted source photons without and with the storage of the gate photon, respectively. In Fig. 4, we show the transistor gain *G* versus *r* = *n*/*N*. From Fig. 4, we see that with the increase of the *r* = *n*/*N*, the optical gain *G* increases firstly and then quickly reaches to the maximum value. Even though *r* ≪ 1, the optical gain *G* can exceed 10^3^. Furthermore, when the cavity is in the weak-coupling regime, i.e., the single-atom cooperativity $\eta ={\bar{g}}^{2}/\kappa {\gamma }_{e}=0.1\ll 1$, the single-photon transistor can still work well.

Then we address the experiment feasibility of the proposed scheme. For a potential experimental system, we consider that an optical cavity traps an ensemble of cold ^87^*Rb* atoms with atom number *N* ≈ 3600. Assuming that *n* ≈ 14 atoms within a blockade sphere with radius *r* ≈ 1.5 *μm* are affected by the Rydberg blockade. For the Rydberg states $|{r}_{1}\rangle =|41{s}_{\mathrm{1/2}},m=\mathrm{1/2}\rangle$, $|{r}_{2}\rangle =|40{s}_{\mathrm{1/2}},m=\mathrm{1/2}\rangle$, one could achieve strongly asymmetric Rydberg blockade interaction ${{\rm{\Delta }}}_{{r}_{1}{r}_{2}}\approx 56$ MHz, which is much larger than ${{\rm{\Delta }}}_{{r}_{2},{r}_{2}}\approx 0.3$ MHz^45,48^. Typically, the relevant cavity parameters are (*κ*, *γ*_*e*_) ≈ (1.16, 37.6) MHz^49^. We choose the parameters *γ*_*r*_ = 10^−2^*κ*, *ε*_*s*_ = 5 MHz, Ω_2_ = 20 MHz, *τ* = 50 *μ*s and the single-atom cooperativity $\eta ={\bar{g}}^{2}/\kappa {\gamma }_{e}=0.1\ll 1$, then we can obtain the optical gain *G* ≈ 1125 for the single-photon transistor. As the experiment progresses, cavity EIT^15^ and multi-wave mixing^50,51^ for strong nonlinearity has been successfully demonstrated in Rydberg atoms. Therefore, our scheme could be realized in the near future.

In conclusion, we have demonstrated a new scheme to implement a single-photon transistor based on cavity QED and Rydberg-EIT. By combining the advantages of the cavity-enhanced interaction and Rydberg blockade, the optical gain of the single-photon transistor is boosted to over 10^3^, which is higher than both cavity scheme^33^ and Rydberg-EIT scheme^34–38^. Furthermore, even when the scale of atomic ensemble is much larger than the blockade radius^44^, our scheme could still work well. Therefore, our work may provide a promising approach for the realization of the single-photon transistor and other all-optical devices.

## Methods

For convenience, we define the collective operators ${S}_{n,{\rm{\Lambda }}}^{\dagger }=\frac{1}{\sqrt{n}}{\sum }_{t=1}^{n}|{{\rm{\Lambda }}}_{t}\rangle \langle {g}_{t}|$ and ${S}_{N-n,{\rm{\Lambda }}}^{\dagger }=\frac{1}{\sqrt{N-n}}{\sum }_{k=1}^{N-n}|{{\rm{\Lambda }}}_{k}\rangle \langle {g}_{k}|$ (Λ = *e*_2_, *r*_2_) for atoms within the Rydberg blockade radius and outside the Rydberg blockade radius, respectively. In terms of these collective operators, we rewrite the Hamiltonians *H*_*n*_ and *H*_*N*−*n*_ as:

9 $${H}_{n}^{^{\prime} }=\sqrt{n}\bar{g}({a}^{\dagger }{S}_{n,{e}_{2}}+a{S}_{n,{e}_{2}}^{\dagger })+{{\rm{\Omega }}}_{2}({S}_{n,{e}_{2}}^{\dagger }{S}_{n,{r}_{2}}+{S}_{n,{r}_{2}}^{\dagger }{S}_{n,{e}_{2}})+{{\rm{\Delta }}}_{{r}_{1},{r}_{2}}{S}_{n,{r}_{2}}^{\dagger }{S}_{n,{r}_{2}},$$

10 $${H}_{N-n}^{^{\prime} }=\sqrt{N-n}\bar{g}({a}^{\dagger }{S}_{N-n,{e}_{2}}+a{S}_{N-n,{e}_{2}}^{\dagger })+{{\rm{\Omega }}}_{2}({S}_{N-n,{e}_{2}}^{\dagger }{S}_{N-n,{r}_{2}}+{S}_{N-n,{r}_{2}}^{\dagger }{S}_{N-n,{e}_{2}}),$$

where $\sqrt{n}\bar{g}=\sqrt{{\sum }_{t=1}^{n}{g}_{t}^{2}}$ $(\sqrt{N-n}\bar{g}=\sqrt{{\sum }_{k=1}^{N-n}{g}_{k}^{2}})$^52^ is the effective atom-cavity coupling strength, which is collectively enhanced due to the many-atom interference effect^53^.

Considering photon losses from the cavity as well as the decays associated with the atom, we describe the dynamics of the system governed by the total Hamiltonian *H*_*total*_ in the rotating frame with the following quantum Langevin equations^46^:

11 $$\dot{a}=-\,(i{{\rm{\Delta }}}_{s}+\kappa )a-i\bar{g}\sqrt{n}{S}_{n,{e}_{2}}-i\bar{g}\sqrt{N-n}{S}_{N-n,{e}_{2}}-i{\varepsilon }_{s},$$

12 $${\dot{S}}_{n,{e}_{2}}=-(i{{\rm{\Delta }}}_{s}+\frac{{\gamma }_{e}}{2}){S}_{n,{e}_{2}}-i\bar{g}\sqrt{n}a-i{{\rm{\Omega }}}_{2}{S}_{n,{r}_{2}},$$

13 $${\dot{S}}_{n,{r}_{2}}=-i(i{{\rm{\Delta }}}_{s}+i{{\rm{\Delta }}}_{{r}_{1},{r}_{2}}+\frac{{\gamma }_{r}}{2}){S}_{n,{r}_{2}}-i{{\rm{\Omega }}}_{2}{S}_{n,{e}_{2}},$$

14 $${\dot{S}}_{N-n,{e}_{2}}=-(i{{\rm{\Delta }}}_{s}+\frac{{\gamma }_{e}}{2}){S}_{N-n,{e}_{2}}-i\bar{g}\sqrt{N-n}a-i{{\rm{\Omega }}}_{2}{S}_{N-n,{r}_{2}},$$

15 $${\dot{S}}_{N-n,{r}_{2}}=-(i{{\rm{\Delta }}}_{s}+\frac{{\gamma }_{r}}{2}){S}_{N-n,{r}_{2}}-i{{\rm{\Omega }}}_{2}{S}_{N-n,{e}_{2}},$$

Here, we have ignored the decoherence between the ground states. Under the mean-field approximation $\langle Qc\rangle =\langle Q\rangle \langle c\rangle$^47^, and the mean value equations are given by

16 $$\langle \dot{a}\rangle =-\,(i{{\rm{\Delta }}}_{s}+\kappa )\langle a\rangle -i\bar{g}\sqrt{n}\langle {S}_{n,{e}_{2}}\rangle -i\bar{g}\sqrt{N-n}\langle {S}_{n,{e}_{2}}\rangle -i{\varepsilon }_{s},$$

17 $$\langle {\dot{S}}_{n,{e}_{2}}\rangle =-\,(i{{\rm{\Delta }}}_{s}+\frac{{\gamma }_{e}}{2})\langle {S}_{n,{e}_{2}}\rangle -i\bar{g}\sqrt{n}\langle a\rangle -i{{\rm{\Omega }}}_{2}\langle {S}_{n,{r}_{2}}\rangle ,$$

18 $$\langle {\dot{S}}_{n,{r}_{2}}\rangle =-\,(i{{\rm{\Delta }}}_{s}+i{{\rm{\Delta }}}_{{r}_{1},{r}_{2}}+\frac{{\gamma }_{r}}{2})\langle {S}_{n,{e}_{2}}\rangle -i{{\rm{\Omega }}}_{2}\langle {S}_{n,{e}_{2}}\rangle ,$$

19 $$\langle {\dot{S}}_{N-n,{e}_{2}}\rangle =-\,(i{{\rm{\Delta }}}_{s}+\frac{{\gamma }_{e}}{2})\langle {S}_{N-n,{e}_{2}}\rangle -i\bar{g}\sqrt{N-n}\langle a\rangle -i{{\rm{\Omega }}}_{2}\langle {S}_{N-n,{r}_{2}}\rangle ,$$

20 $$\langle {\dot{S}}_{N-n,{r}_{2}}\rangle =-\,(i{{\rm{\Delta }}}_{s}+\frac{{\gamma }_{r}}{2})\langle {S}_{N-n,{r}_{2}}\rangle -i{{\rm{\Omega }}}_{2}\langle {S}_{N-n,{e}_{2}}\rangle ,$$

The steady-state solution of the cavity mode is given by

21 $$\langle a\rangle =\frac{-i{\varepsilon }_{s}}{i{{\rm{\Delta }}}_{s}+\kappa +\frac{{\bar{g}}^{2}n}{i{{\rm{\Delta }}}_{s}+\frac{{\gamma }_{e}}{2}+\frac{{{\rm{\Omega }}}_{2}^{2}}{i{{\rm{\Delta }}}_{s}+i{{\rm{\Delta }}}_{{r}_{1},{r}_{2}}+\frac{{\gamma }_{r}}{2}}}+\frac{{\bar{g}}^{2}(N-n)}{i{{\rm{\Delta }}}_{s}+\frac{{\gamma }_{e}}{2}+\frac{{{\rm{\Omega }}}_{2}^{2}}{i{{\rm{\Delta }}}_{s}+\frac{{\gamma }_{r}}{2}}}}\mathrm{.}$$

## Data Availability

All data generated or analysed during this study are included in this published article (and its Supplementary Information files).

## References

[1] Imamoglu A, Schmidt H, Woods G, Deutsch M (1998). Strongly interacting photons in a nonlinear Cavity. Phys. Rev. Lett..

[2] Schmidt H, Imamoglu A (1996). Giant Kerr nonlinearities obtained by electromagnetically induced transparency. Opt. Lett..

[3] Fleischhauer M, Lukin MD (2000). Dark-state polaritons in electromagnetically induced transparency. Phys. Rev. Lett..

[4] Gorshkov AV, André A, Lukin MD, Sørensen AS (2007). Photon storage in Λ-type optically dense atomic media. I. Cavity model. Phys. Rev. A.

[5] Zhang ZY (2018). M. Non-Hermitian optics in atomic systems. J. Phys. B: At. Mol. Opt. Phys..

[6] Zhang ZY (2018). Parity-time-symmetric optical lattice with alternating gain and loss atomic configurations. Laser Photon. Rev..

[7] Zhang ZY (2016). Observation of parity-time symmetry in optically induced atomic lattices. Phys. Rev. Lett..

[8] Zhang ZY (2018). Controllable photonic crystal with periodic Raman gain in a coherent atomic medium. Opt. Lett..

[9] Zhang Y, Wen JM, Zhu SN, Xiao M (2010). Nonlinear Talbot effect. Phys. Rev. Lett..

[10] Parigi V (2012). Observation and measurement of interaction-induced dispersive optical nonlinearities in an ensemble of cold Rydberg atoms. Phys. Rev. Lett..

[11] Boddeda R (2016). Rydberg-induced optical nonlinearities from a cold atomic ensemble trapped inside a cavity. J. Phys. B: At. Mol. Opt. Phys..

[12] Grankin A (2014). Quantum statistics of light transmitted through an intracavity Rydberg medium. New J of Phys.

[13] Grankin A (2015). Quantum-optical nonlinearities induced by Rydberg-Rydberg interactions: A perturbative approach. Phys. Rev. A.

[14] Lin GW, Qi YH, Lin XM, Niu YP, Gong SQ (2015). Strong photon blockade with intracavity electromagnetically induced transparency in blockaded Rydberg ensemble. Phys. Rev. A.

[15] Jia N (2018). A strongly interacting polaritonic quantum dot. Nat. Phys..

[16] Hao YM (2015). Quantum controlled-phase-flip gate between a flying optical photon and a Rydberg atomic ensemble. Sci. Rep..

[17] Das S (2016). Photonic controlled-phase gates through Rydberg blockade in optical cavities. Phys. Rev. A.

[18] Caulfield HJ, Dolev S (2010). Why future supercomputing requires optics. Nat. Photonics.

[19] O’Brien JL, Furusawa A, Vučković J (2009). Photonic quantum technologies. Nat. Photonics.

[20] Bermel P, Rodriguez A, Johnson SG, Joannopoulos JD, Soljačić M (2006). Single-photon all-optical switching using waveguide-cavity quantum electrodynamics. Phys. Rev. A.

[21] Chang DE, Sørensen AS, Demler EA, Lukin MD (2007). A single-photon transistor using nanoscale surface plasmons. Nat. Phys..

[22] Nielsen, M. A. & Chuang, I. L. Quantum computation and quantum information (University Press, Cambridge, 2000).

[23] Briegel H-J, Dür W, Cirac JI, Zoller P (1998). Quantum repeaters: the role of imperfect local operations in quantum communication. Phys. Rev. Lett..

[24] Braginsky VB, Khalili F (1996). Ya. Quantum nondemolition measurements: the route from toys to tools. Rev. Mod. Phys..

[25] Gheri KM, Ritsch H (1997). Single-atom quantum gate for light. Phys. Rev. A.

[26] Hwang J (2009). A single-molecule optical transistor. Nature.

[27] Bajcsy M (2009). Efficient all-optical switching using slow light within a hollow fiber. Phys. Rev. Lett..

[28] Englund D (2012). Ultrafast photon-photon interaction in a strongly coupled quantum dot-cavity system. Phys. Rev. Lett..

[29] Bose R, Sridharan D, Kim H, Solomon GS, Waks E (2012). Low-photon-number optical switching with a single quantum dot coupled to a photonic crystal cavity. Phys. Rev. Lett..

[30] Volz T (2012). Ultrafast all-optical switching by single photons. Nat. Photonics.

[31] Loo V (2012). Optical nonlinearity for few-photon pulses on a quantum dot-pillar cavity device. Phys. Rev. Lett..

[32] Arkhipkin VG, Myslivets SA (2013). All-optical transistor using a photonic-crystal cavity with an active Raman gain medium. Phys. Rev. A.

[33] Chen W (2013). All-optical switch and transistor gated by one stored photon. Science.

[34] Baur S, Tiarks D, Rempe G, Dürr S (2014). Single-photon switch based on Rydberg blockade. Phys. Rev. Lett..

[35] Neumeier L, Leib M, Hartmann MJ (2013). Single-photon transistor in circuit quantum electrodynamics. Phys. Rev. Lett..

[36] Gorniaczyk H, Tresp C, Schmidt J, Fedder H, Hofferberth S (2014). Single-photon transistor mediated by interstate Rydberg interactions. Phys. Rev. Lett..

[37] Tiarks D, Baur S, Schneider K, Dürr S, Rempe G (2014). Single-photon transistor using a Förster resonance. Phys. Rev. Lett..

[38] Gorniaczyk H (2016). Enhancement of Rydberg-mediated single-photon nonlinearities by electrically tuned Förster resonances. Nat Communications.

[39] Zhang ZY (2015). Phase modulation in Rydberg dressed Multi-Wave mixing processes. Sci. Rep..

[40] Firstenberg O, Adams CS, Hofferberth S (2016). Nonlinear quantum optics mediated by Rydberg interactions. J. Phys. B: At. Mol. Opt. Phys..

[41] Gorshkov AV, Otterbach J, Fleischhauer M, Pohl T, Lukin MD (2011). Photon-photon interactions via Rydberg blockade. Phys. Rev. Lett..

[42] Sheng JT (2017). Intracavity Rydberg-atom electromagnetically induced transparency using a high-finesse optical cavity. Phys. Rev. A.

[43] Jia N (2016). Observation and characterization of cavity Rydberg polaritons. Phys. Rev. A.

[44] Guerlin C, Brion E, Esslinger T, Mølmer K (2010). Cavity quantum electrodynamics with a Rydberg-blocked atomic ensemble. Phys. Rev. A.

[45] Saffman M, Mølmer K (2009). Efficient multiparticle entanglement via asymmetric Rydberg blockade. Phys. Rev. Lett..

[46] Walls, D. F. & Milburn, G. J. *Quantum Optics* (Springer-Verlag, Berlin, 1994).

[47] Agarwal GS, Huang S (2010). Electromagnetically induced transparency in mechanical effects of light. Phys. Rev. A.

[48] Walker TG, Saffman M (2007). Consequences of Zeeman degeneracy for the van der Waals blockade between Rydberg atoms. Phys. Rev. A.

[49] Tanji-Suzuki H, Chen W, Landig R, Simon J, Vuletić V (2011). Vacuum-induced transparency. Science.

[50] Che JL, Zhang ZY, Hu ML, Shi XW, Zhang YP (2018). Novel Rydberg eight-wave mixing process controlled in the nonlinear phase of a circularly polarized field. Opt. Express..

[51] Che JL (2016). Polarized Autler–Townes splitting of Rydberg six-wave mixing. J. Phys. B: At. Mol. Opt. Phys..

[52] Mücke M (2010). Electromagnetically induced transparency with single atoms in a cavity. Nature.

[53] Lange W (2009). Cavity QED: Strength in numbers. Nat. Phys..

